# Constriction of the Stomach by an Unusual Peritoneal Band

**DOI:** 10.7759/cureus.2148

**Published:** 2018-02-03

**Authors:** Mohammad W Kassem, Mayank Patel, Joe Iwanaga, Marios Loukas, R. Shane Tubbs

**Affiliations:** 1 Clinical Anatomy Research, Seattle Science Foundation; 2 Seattle Science Foundation; 3 Department of Anatomical Sciences, St. George's University School of Medicine, Grenada, West Indies; 4 Neurosurgery, Seattle Science Foundation

**Keywords:** peritoneal bands, omentum, ladd's bands, stomach constriction

## Abstract

Compression of intraabdominal contents can occur due to anomalous congenital bands. Herein, we describe, to our knowledge, the first case of compression of the stomach by an anomalous band extending from the lesser omentum to the greater omentum. Relevant literature is reviewed and the clinical implications of such a case are described.

## Introduction

Peritoneal bands, or Ladd’s bands, are fibrous stalks of the peritoneum that develop aberrantly during errors in mesenteric development. They have long been discussed in the literature and can have significant clinical consequences. In 1901, Sir Arbuthnot Lane gave what are now known to be descriptions of peritoneal bands.

Reviewing the embryology of the gut is crucial for understanding where and why peritoneal bands form. The primitive gut begins to form during the fourth week of embryonic development, and, by the fifth week, the vascular supply dictates the division into foregut, midgut, and hindgut. In stage one, the embryo undergoes cephalocaudal and lateral folding, focusing on the midgut, which forms the primary intestinal loop from the ligament of Treitz to the proximal two-thirds of the transverse colon. As the sixth-week approaches, the loop continues to grow and becomes larger than the space containing it. Eventually, the loop herniates out of the extraembryonic cavity. The loop returns into the cavity by the tenth week. During stage two, weeks 10-11, after the herniation returns, the midgut loop rotates 270° counterclockwise, and the portion that becomes the ascending colon begins to rotate to the right. The third and final stage is the period of fixation, which begins when stage two ends and continues until shortly after birth [1]. During stage two of gut development, multiple anomalies can occur including the failure of the midgut to return to the abdominal cavity, stenosis, replication of parts, and malrotation, and remnants of the vitelline duct can persist.

The gut, during this portion of the development, is suspended by the mesentery, a double-layered serous membrane that encloses the gastrointestinal tract between its two enveloping layers. As the mesentery develops, it is split into dorsal and ventral portions, the former containing the pancreas, spleen, gut, lymphatic, and neurovascular vessels originating from and traveling to various visceral organs. The latter, the ventral portion of the mesentery, encloses the liver, stomach, and the duodenal portion of the small intestine. Once fetal development has concluded, embryonic forms of the mesenteries are normally obliterated, but sometimes remnants of the primitive mesenteries persist and give rise to peritoneal bands. These are commonly found in the duodenojejunal flexure, the terminal portion of the ileum, the cecal region, the duodenum, the ascending and descending colon, and the sigmoid colon [2].

Aberrations in the various stages of gut development lead to specific clinical problems. Stage one errors, where the failure of the gut tube to rotate properly around the mesenteric axis, cause the duodenojejunal junction to become located anterior (instead of posterior, as in normal development) to the superior mesenteric artery (SMA). In consequence, the cecum is not located in the right lower quadrant. As a result of this aberration and the physical “relocation” of the cecum, the mesentery becomes short and narrow, twisting in on itself, causing midgut volvulus. Stage two errors caused by incomplete rotation permit the formation of peritoneal bands caused by the out-of-place mesentery and intestine, leading to internal herniation, intestinal obstructions, and volvulus [3].

With such complex folds and intricate detail in timing, the clinical implications can be significant and serious. Aberrations of the gut formation during embryogenesis can have results ranging from major clinical repercussions to no clinical signs. Congenital malrotation is present in 80% of cases within the first four weeks of the newborn’s life [4].

Peritoneal bands often travel from the cecum to the right upper quadrant of the retroperitoneum, leading to entrapment of the transverse and descending portions of the duodenum. These bands can cause different degrees of obstruction, almost always leading to a small bowel obstruction (SBO). According to some accounts, up to 3% of all intestinal obstructions are caused by congenital peritoneal bands [5]. Most peritoneal bands are associated with malrotation of the intestine, with and without midgut volvulus, and are said to occur in one in 500 newborns [4].

When malrotation of the small bowel mesentery occurs around the SMA, the mesenteric base is narrowed, whereupon Ladd’s bands cause obstruction, volvulus, or simply duodenal stasis [2]. Symptoms present differently in older and younger children. Older children often have chronic abdominal pains that wax and wane. However, when the intermittent obstruction becomes a sustained acute obstruction or volvulus, the clinical picture changes. Acute symptoms of nausea and bilious vomiting are the first clinical signs to note [6]. Universal symptoms can occur, such as vague abdominal pain that the patient cannot localize to one region of the abdomen. Patients can complain of bloating, constipation or diarrhea, abdominal distension and tenderness to palpation, sensation of colicky pain, and even malabsorption [7].

Here, we report a very unusual finding where a peritoneal band was found to compress the stomach. Salient literature is discussed and the clinical implications are reviewed.

## Case presentation

**Figure 1:**
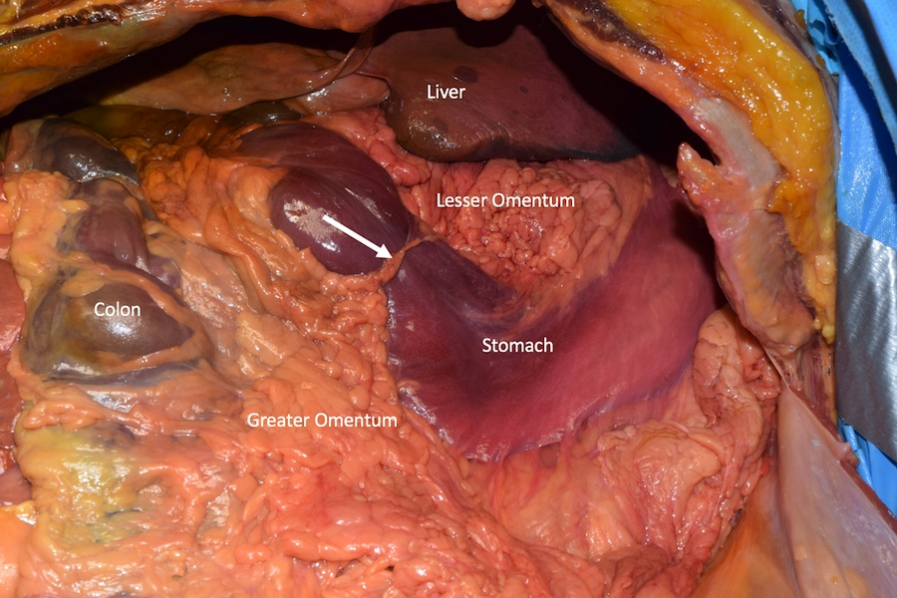
Anterior dissection of the abdominal cavity Note the anomalous band (arrow) compression of the anterior stomach, traveling between the lesser omentum and greater omentum.

During routine dissection within the abdominal cavity of an 87-year-old female cadaver, a distinct constriction of the distal stomach proximal to the pylorus was observed (Figure 1). The constricting band extended from the lesser omentum (hepatogastric ligament) to the greater omentum. This fat laden band was 6 cm in length and 1.4 cm in width at its widest point and 5 mm at its narrowest point. There was no history of abdominal surgery or trauma to the abdomen. The cause of death was heart failure. Further dissection of the abdominal cavity found no other gross pathology or anatomical variations. Mild distension of the pylorus could be seen.

## Discussion

Based on literature review, gastric constriction by a peritoneal band has not been previously reported. Although Finech et al. reported strangulation of the stomach by a band traveling from the mesentery to the diaphragm [8], it is unlike the band from the lesser to greater omentum as seen in our cadaveric specimen but relatable.

Diagnosing peritoneal anomalies is difficult because there are relatively few external indicators that a provider can identify by clinically looking at a patient with suspected peritoneal aberrations. The best approach is to begin with a plain radiograph, which allows dilated bowel loops and multiple air-fluid levels to be visualized. Barium studies are the gold standard for imaging obstructions. Contrast studies can identify obstructions within the small intestine and even identify misplaced intestines in the abdomen, which can suggest peritoneal band aberrations. Small bowel follow-through will also visualize the size of the obstruction by displaying dilated loops or delay in barium transit. Computed tomography (CT) is generally used for patients suspected of an acute obstruction, due to quick procedure time and non-invasiveness. If an obstruction with ischemic changes is suspected due to strangulation, a contrasted CT will allow perfusion anomalies to be visualized more clearly [9].

Treatment of a peritoneal band anomaly is crucial for preventing bowel obstruction, volvulus, herniation, strangulation, or necrosis of the bowel. Ladd’s procedure is the definitive treatment in children and in adults for some time now. It is crucial to diagnose and treat malrotation as soon as possible to avoid midgut volvulus. It consists of surgically removing the bowel and untwisting the volvulus in a counterclockwise direction. The surgeon also separates the Ladd’s bands and mobilizes the cecum, allowing the small bowel to be placed in the right paracolic gutter and the large bowel into the left colic gutter [10].

## Conclusions

To our knowledge, compression of the stomach due to an anomalous peritoneal band has not previously been reported. Although rare, such an anomaly should be considered by physicians in cases of suspected gastrointestinal obstruction.
